# 

**Published:** 1900-05-05

**Authors:** 


### NOTICE TO CORRESPONDENTS.

All MS., Letters, Books for Review, and other matters intended for the Editor should be addressed The Editor, "The Hospital," The Hospital Building, 28 & 29, Southampton Street, Strand, W.C.

The Editor cannot undertake to return rejected MS., even when accompanied by stamped directed envelope.

**Notice to Advertisers and Subscribers.**

All Advertisements, Orders for Copies of The Hospital, and Business Communications should be addressed to **THE MANAGER (Not to the Editor)**, The Hospital, The Hospital Building, 28 & 29, Southampton Street, Strand, London, W.C.

The Cost of Subscription, post free, is as follows:—Per week, 2½d.; per quarter, 2s. 8d.; per half-year, 5s. 3d.; per annum, 10s. 6d. Foreign:—Per annum, 15s. 2d., payable, in all cases, in advance.

**NOTICE.—Vol. XXVII. of "The Hospital" (October, 1899, to April, 1900), in handsome cloth binding, will shortly be on sale at the Office of this Journal, price 7s. 6d. Cases for binding the Numbers contained in this Volume 1s. 6d. Index to Vol. XXVII., 2½d., post free.**

**The Monthly Part for May is now ready. Price 6d., by post 8d.**

### SCRAPS AND GLEANINGS.

Lord Durham has consented to become President of the Durham County Hospital.

An amateur dramatic entertainment in aid of the Cheltenham Eye, Ear, and Throat Hospital was recently given.

The Metropolitan Hospital, Kingsland Road, N.E., has received a donation of £300 from the Goldsmiths' Company.

Lady Wimborne has given a donation of £700 to wipe off the deficit on last year's working of the Cornelia Hospital, Poole.

The final plans and estimates for the new nurses' home of the Norfolk and Norwich Hospital, for which the munificent sum of £15,000 has been given by the Earl of Leicester, are now in hand.

The new cottage hospital for Ramsbottom, the gift of Mr. and Mrs. Aitken, provides accommodation for eight patients in two wards of four beds each. The property is vested in nine trustees, and the institution is to be supported by voluntary contributions.

The Central Hackney Musical and Benevolent Society, the object of whose existence is to assist the North-Eastern Hospital for Children, recently gave its first annual dinner, which proved a great success. Donations amounting to more than £300 were announced.

The amount received by the Bristol Hospital Sunday Fund as a result of the 1899 collections was £781, from 97 churches which made a collection; 113 chapels produced £580 odd, so that the total collection was £1,361 odd, the expenses of the fund amounting to £39 odd.

The Ongar Rural District Council, finding it difficult, if not impossible, to secure an offer so as to purchase the land required for the erection of an isolation hospital, have applied to the Local Government Board asking them to issue an order empowering them to compulsorily acquire the necessary site.

At the recent half-yearly meeting of the Derbyshire Royal Infirmary the resolution empowering the board to obtain and accept tenders for the erection of an isolated block for septic and observation cases was unanimously carried. This addition is estimated to cost an outside and inclusive sum of £6,000. This new block will contain accommodation for seven beds for septic and two beds for observation cases.

The enlarged building of the Bristol Eye Hospital is rapidly approaching completion. The improvements and alterations include the addition of an entirely new storey to provide proper sleeping accommodation for the nurses and servants, some small wards for patients, and a good operating theatre. A new wing is also being built which will provide, among other accommodation, a new operating theatre for out-patient cases.

The North Riding Infirmary are urgently appealing for further and increased subscriptions to avoid in future the financial difficulties in which they are at present, as their indebtedness at the close of the year amounts to £1,061. The building extension scheme is nearing completion; all the amounts on this account have been paid for with the exception of £1,444, of which the committee have on hand a sum of £885, thus leaving £559 to be subscribed.

The appeal to the public by the Norfolk and Norwich Hospital, and the knowledge that unless increased funds were forthcoming the work of the hospital would have to be seriously curtailed, has been productive of satisfactory results. At the recent annual meeting it was stated that the subscriptions had already been increased by £800. The minimum sum required in annual income to enable the hospital to continue its work efficiently is £4,000, and it is to be hoped that henceforward such a sum will be forthcoming.

Dean Farrar and Admiral Field, C.B., M.P., spoke at Exeter Hall on April 24th on the occasion of the fifty-seventh annual meeting of the National Refuges and Training Ships "Arethusa" and "Chichester."

In consequence of the report of the architect on St. Bartholomew's Hospital, Chatham, the trustees find it is necessary to make immediate provision for (1) a new operating theatre; (2) a lift to raise patients from the receiving rooms to the level of the theatre and the various wards; (3) a children's ward.

The special committee appointed to report, after consultation with an expert, on the future administration of the Tunbridge Wells General Hospital, have issued their report and that of Mr. P. Michelli, the expert consulted. Mr. Michelli goes fully into various defects, and the special committee generally endorse his report and recommend its adoption, with a few additional suggestions by themselves.

### THE STUDENT OF MEDICINE.

### APPOINTMENTS.

N. W. Anderson, M.B., C.M.Aberd., appointed Certifying Factory Surgeon for the Civil Parish of Strathmiglo, co. Fife. G. W. H. Bird, M.A., M.B., B.C.Cantab., M.R.C.S., L.R.C.P., appointed Surgeon to the Bridgwater Infirmary. C. R. Box, M.D., B.S., B.Sc.Lond., M.R.C.P., L.R.C.S., appointed Assistant Physician to St. Thomas's Hospital. A. Brown, M.R.C.S., appointed Medical Officer for the Masham District of the Bedale Union. W. J. Caie, M.A., M.B., Ch.B., appointed Resident Physician and Surgeon to the Aberdeen Royal Infirmary. R. Craven, M.B., appointed Medical Officer for the Chipping District of the Clitheroe Union. T. S. Hearder, M.B., C.M.Edin., appointed Certifying Surgeon for the Urban District of Ilkley, and the Townships of Nesfield-with-Langbar, Middleton, Denton, Askwith, Great Timble, Little Timble, Blubberhouses, and Fewston. C. E. Hollings, L.R.C.P., L.R.C.S.Edin., appointed Medical Officer of Health for the Driffield Rural District. W. D. Jones, L.R.C.P., L.R.C.S.E., L.S.A.Lond., appointed Medical Officer and Public Vaccinator to the Rotherham No. 1 District. J. F. Macpherson, M.B., C.M.Edin., appointed Medical Officer for the First District of the Basingstoke Union. D. Massey, L.R.C.P., L.R.C.S.I., appointed Medical Officer for the Berriew District of the Forden Union. H. Peterkin, M.B., Ch.B., appointed Resident Physician and Surgeon to the Aberdeen Royal Infirmary. H. Sharp, M.B., appointed Medical Officer of Health to the Corporation of Truro. H. J. Spon, M.R.C.S.Eng., D.P.H.-Camb., appointed Visiting Medical Officer to the City Dispensary, College Street, E.C. J. D. Walker, M.B., appointed Medical Officer for the Fontmell District of the Shaftesbury Union. L. H. Williams, M.R.C.S.Eng., appointed Certifying Factory Surgeon for the Civil Parishes of Thornbury, Oldbury-upon-Severn, Littleton upon-Severn, Rockhampton, Falfield, Tortworth, Charfield, Cromhall Abbotts and Cromhall Lygon, Rangeworthy, and Tytherington in the Thornbury Rural District. J. M. S. Wood, M.B., Ch.B., appointed Assistant Medical Officer at James Murray's Royal Asylum, Perth.

Demy 8vo., with Portrait, cloth gilt, 16s.

GEORGE HARLEY, F.R.S.,

Or, The Life of a London Physician.

By Mrs. ALEC TWEEDIE.

"The authoress has succeeded, by a judicious combination of her father's notes with her own recollections, in producing a readable and interesting memoir."—*The Times.*

London: The Scientific Press, Limited, 28 & 29, Southampton Street, Strand, W.C.

"The Prototype of all Bitter Waters."—Lancet.

**Hunyadi János**

"Speedy, sure and gentle."—Brit. Med. Jour., 1884.

"Speedy, sure and gentle."—Brit. Med. Jour., 1890.

"Speedy, sure and gentle."—Practitioner, 1896.

PROPRIETOR:

ANDREAS SAXLEHNER, BUDAPEST.

### BOOKS FOR NURSES.

**Domville's Manual for Hospital Nurses**, 8th edition ... ... ... ... ... ... ... ... **2/6**
**Cullingworth's Monthly Nursing**, 4th edition... **1/6**
**Hellier's Gynæcological Nursing**... ... ... **1/6**
**Cuff's Lectures on Medicine to Nurses**... ... **3/6**
**Richmond's Antiseptic Principles for Nurses** **1/-**
**Mayne's Short Dictionary of Medical Terms**, **2/6**
**Chavasse's Advice to a Wife**, 14th edition, by Dr. FANCOURT BARNES ... ... ... ... **2/6**
**Chavasse's Advice to a Mother**, 15th edition, by Dr. GEORGE CARPENTER .. ... ... ... **2/6**

LONDON: J. & A. CHURCHILL, 7, Great Marlborough Street.

### THE NURSE'S REPORT BOOK.

Foolscap 4to., Black cover, **6d.** net. By Post 8d.

**Compiled by K. H. (Cert.)**

CONTAINING space for general details at the beginning of the book, and for general remarks on the case at the end of the book, and with space for 200 entries of each of the following details: **Time—Nourishment—Stimulants—Temperature—Pulse—Respiration—Urine—B.O.—Sleep—Remarks.**

H. J. GLAISHER, 57, Wigmore Street, Cavendish Square, W.

### NOW READY,

A REPRINT OF

### A NURSING HANDBOOK

TOGETHER WITH S. MAW, SON, AND THOMPSON'S

### COMPLETE CATALOGUE OF NURSING REQUISITES.

The above work is now republished at a cost of 1s. per copy. Messrs. S. M., S., and T. have been compelled to make this charge on account of the extreme popularity of the work and the great expense incurred in its production.

***Post Free on receipt of 1s.***

### S. MAW, SON, and THOMPSON,

7 to 12, ALDERSGATE STREET, LONDON, E.C.

